# Clinical Characteristics and Outcome of Children Hospitalized With Scrub Typhus in an Area of Endemicity

**DOI:** 10.1093/jpids/piz014

**Published:** 2019-03-13

**Authors:** Tri Wangrangsimakul, Rachel C Greer, Chulapong Chanta, Supalert Nedsuwan, Stuart D Blacksell, Nicholas P J Day, Daniel H Paris

**Affiliations:** 1 Mahidol-Oxford Tropical Medicine Research Unit, Faculty of Tropical Medicine, Mahidol University, Bangkok, Thailand; 2 Centre for Tropical Medicine and Global Health, Nuffield Department of Clinical Medicine, University of Oxford, United Kingdom; 3 Department of Pediatrics, Chiang Rai Prachanukroh Hospital, Thailand; 4 Department of Family Medicine, Chiang Rai Prachanukroh Hospital, Chiang Rai, Thailand; 5 Department of Medicine, Swiss Tropical and Public Health Institute and Faculty of Medicine, University of Basel, Switzerland

**Keywords:** children, *Orientia tsutsugamushi*, outcomes, scrub typhus, Thailand

## Abstract

**Background:**

Scrub typhus, caused by *Orientia tsutsugamushi*, is a major cause of acute febrile illness in children in the rural tropics.

**Methods:**

We recruited 60 febrile pediatric patients with a positive scrub typhus rapid diagnostic test result and 40 healthy controls from Chiang Rai Province in northern Thailand. Diagnosis was confirmed by the detection of (1) *O. tsutsugamushi–*specific DNA in blood or eschar samples with a polymerase chain reaction assay, (2) a fourfold rise in immunoglobulin M (IgM) titer to ≥1:3200 in paired plasma samples with an indirect immunofluorescence assay (IFA), or (3) a single IgM titer of ≥1:3200 in an acute plasma sample with an IFA. Demographic, clinical, and laboratory data were collected, and patients were followed up for 1 year.

**Results:**

Diagnosis was confirmed in 35 (58%) of 60 patients, and all controls tested negative for scrub typhus. Patients with confirmed scrub typhus had clinical symptoms, including fever (35 of 35 [100%]), eschar (21 of 35 [60%]), cough (21 of 35 [60%]), tachypnea (16 of 35 [46%]), lymphadenopathy (15 of 35 [43%]), and headache (14 of 35 [40%]). Only 4 (11%) of 35 patients received appropriate antibiotic treatment for scrub typhus before admission. The median fever-clearance time was 36 hours (interquartile range, 24–53 hours). Complications observed include hepatitis (9 of 35 [26%]), severe thrombocytopenia (7 of 35 [20%]), pneumonitis (5 of 35 [14%]), circulatory shock (4 of 35 [11%]), and acute respiratory distress syndrome (3 of 35 [9%]). Treatment failure, defined by failure to defervesce within 72 hours of antibiotic treatment initiation, was noted in 8 (23%) of 35 patients, and 1 (3%) of the 35 patients died. No evidence of relapse or reinfection was found.

**Conclusion:**

Pediatric scrub typhus in northern Thailand is often severe and potentially fatal with delays in treatment a likely contributing factor. Additional studies to investigate the bacterial, pharmacologic, and immunologic factors related to treatment outcome along with measures to improve public awareness should be prioritized.

## Teaser

Scrub typhus is a major cause of acute febrile illness in the tropics. Pediatric scrub typhus in northern Thailand is often severe and potentially fatal, and it is compounded by challenges surrounding its diagnosis, treatment, and lack of awareness.

Scrub typhus, a potentially severe but treatable infection caused by the obligate intracellular bacterium *Orientia tsutsugamushi*, is a major cause of acute nonmalarial febrile illness in children in the rural tropics [1–3]. Transmission occurs when trombiculid mites, in their larval stage, feed on humans [4]. The disease is endemic over an area of at least 13,000,000 km^2^ of the Asia Pacific region although recent reports suggest it is much more widespread [5, 6]. Historically, the burden of scrub typhus in children has been high. The proportion of pediatric cases ranged from 50% to 74% in southern China and Taiwan, respectively, while 52% of children were found to be seropositive in central Thailand [7–9]. Children are less likely to have preexisting immunity and are at greater risk of developing severe disease [10, 11].

The treatment of scrub typhus involves the use of an antibiotic such as doxycycline, chloramphenicol, azithromycin, or rifampicin. A rapid response, signified by fever resolution, was understood to be characteristic of the disease. However, reports have emerged from studies in northern Thailand in which delayed defervescence and severe disease were observed in both adults and children despite treatment with appropriate antibiotics [12, 13]. This finding contrasts with those of 2 other pediatric studies in the same region in which doxycycline and chloramphenicol were uniformly effective in treating scrub typhus [14, 15]. Although these findings have been attributed to drug resistance, supportive evidence is lacking, and the determinants of treatment outcome remain uncharacterized.

Studies of pediatric scrub typhus in which stringent diagnostic criteria were used are scarce. The lack of access to diagnostic tests, such as the gold-standard indirect immunofluorescence assay (IFA), polymerase chain reaction (PCR) assay, and *in vitro* culture, have contributed to this scarcity. Recent studies from the endemic area are limited by their retrospective nature, insufficient follow-up, lack of a control group, heterogeneity in the diagnostic criteria used, and frequent reliance on inadequately validated diagnostic assays [14–20].

All of these assays have inherent weaknesses [21]. The perceived accuracy of serological tests (eg, enzyme-linked immunosorbent assays, IFAs) depend on the antigenic complement of the assay with the infecting bacterial strain and the extent of residual antibodies in the population within areas of endemicity [22, 23]. PCR assays and culture are specific, but their sensitivity can vary, depending on the quality of the samples, disease time point, and bacterial load [23]. Moreover, the limited validation studies performed to date have been focused on adult samples, which results in a degree of uncertainty regarding the accuracy of these assays for children.

In this study, we aimed to characterize pediatric scrub typhus and explore the determinants of treatment outcome in a region for which it was reported to be suboptimal. We investigated the use of robust and validated diagnostic criteria that had not been applied to children previously. To achieve these goals, a prospective observational study in children with scrub typhus in northern Thailand was performed, incorporating a prolonged follow-up period and healthy control group from the same region.

## MATERIALS AND METHODS

### Ethics and Setting

Ethical approval for this study was obtained from the ethics committees of Chiang Rai Prachanukroh Hospital and Chiang Rai Provincial Public Health Office, Ministry of Public Health (Thailand), Faculty of Tropical Medicine (Mahidol University, Bangkok, Thailand), and University of Oxford (Oxford Tropical Research EC, Oxford, United Kingdom). This study was registered at ClinicalTrials.gov (identifier NCT02398162).

Chiang Rai Prachanukroh Hospital is located in Chiang Rai Province, the northernmost province of Thailand bordering Myanmar and Laos. It is the main provincial hospital with a defined catchment area but also accepts patients from outlying healthcare facilities who require escalation of care. The province has a high burden of scrub typhus; the population of 1.25 million consists predominantly of ethnic Thais, and 12% to 13% belong to a hill tribe or other minority ethnic group [24].

### Patients, Study Schedule, and Sample Collection

From July 2015 to August 2016, we prospectively recruited 60 children aged <18 years who were admitted to Chiang Rai Prachanukroh Hospital with fever (temperature > 37.5°C) or history of fever within the preceding 14 days, had a positive scrub typhus immunoglobulin M (IgM) rapid diagnostic test (RDT) result, were not diagnosed with or being treated for tuberculosis, were not immunocompromised, and were not pregnant. Written informed consent was obtained from the parent or guardian, as was the child’s assent if he or she was aged ≥7 years. Demographic, clinical, and laboratory data were collected individually on study case-record forms. Findings from chest radiography (CXR), if performed, were recorded. Fever-clearance time (FCT) was defined as the time taken from the initiation of appropriate antibiotic treatment (doxycycline, chloramphenicol, tetracycline, azithromycin, or rifampicin) to defervescence (temperature ≤ 37.5°C) with the temperature remaining at ≤37.5°C for ≥24 hours after that point. Treatment failure was defined as an FCT of greater than 72 hours after the initiation of appropriate antibiotic treatment. Relapse or reinfection was defined as a return of fever and other symptoms compatible with scrub typhus along with confirmatory laboratory diagnosis, as described below.

Blood samples were collected at baseline and at the 2-, 12-, and 52-week follow-up time points. Blood was collected in ethylenediaminetetraacetic acid (EDTA) and clotted-blood tubes and processed to obtain aliquots of whole blood, plasma, buffy coat, and serum. If present, an eschar swab or crust was collected in 95% ethanol. Samples were stored at −80°C and transported to Bangkok for diagnostic processing. During the follow-up period, patients were also clinically assessed for additional illnesses, and their treatment was recorded. Recruitment and follow-up were completed by August 2017.

During the study period, we also recruited 40 children from the community to act as controls. These children were either siblings of patients or children who were living in areas in which scrub typhus cases had occurred. All of them were healthy, none reported a past history of scrub typhus infection, and almost all of them had no knowledge of the disease. Written informed consent and assent were obtained. Demographic data and blood samples were collected for diagnostic, hematology, and biochemistry tests.

### Diagnostic Assays and Attribution of Diagnosis

The scrub typhus Detect IgM rapid test (InBios International, Inc, Seattle, Washington), an immunochromatographic-based test that uses recombinant 56-kDa type-specific antigen (TSA) of the Karp, Kato, Gilliam, and TA716 strains of *O. tsutsugamushi*, was used for screening [25]. The gold-standard IFA was used to detect IgM antibody titers in paired plasma samples (or in admission samples, only when convalescent samples were unavailable) against pooled *O. tsutsugamushi* antigens from the Karp, Kato, Gilliam, and TA716 strains, as previously described [26]. A validated diagnostic IFA cutoff titer of ≥1:3200 in a single acute-phase sample or a greater than fourfold rise to ≥1:3200 in a convalescent-phase sample was used to determine positivity [27].

*O. tsutsugamushi–*specific PCR assays were performed as previously described [28–30]. In brief, DNA extraction from whole blood, buffy coat, or noninvasive eschar samples (if applicable) was performed. Then, a real-time PCR assay was performed to detect the 47-kDa *htra* gene in duplicate and, if the result was positive, the nested 56-kDa TSA gene PCR assay was performed.

In controls, PCR assays were performed as already described, and serological testing included a screening enzyme-linked immunosorbent assay with a ≥0.5 net optical density cutoff followed by the IFA as appropriate [31]. The diagnosis of scrub typhus was made on the basis of robust criteria previously described and updated with the following validated IFA cutoff titers: (1) a positive PCR or culture result from either a blood or eschar sample, (2) a fourfold rise in the IgM titer to ≥1:3200 in paired serum or plasma samples, or (3) a single IgM titer of ≥1:3200 in an acute-phase serum or plasma sample [27, 32]. Diagnostic PCR and serology assays were performed in the main laboratory facilities at Mahidol-Oxford Tropical Medicine Research Unit in Bangkok, Thailand.

### Statistical Analysis

Proportions, percentages, and averages were calculated (medians and interquartile ranges [IQRs], unless specified). Comparisons of demographic, exposure, and laboratory variables between the patient and control groups were performed using the Mann-Whitney U test, the Pearson’s χ^2^ test, or the Fisher’s exact test, as appropriate. Seasonality was assessed by calculating proportions and their 95% confidence interval (CI) and performing 2-sample tests of proportions. FCTs were plotted using a Kaplan–Meier survival curve. Treatment failure was analyzed using logistic regression. Analyses were performed using Stata 15 software (College Station, Texas).

## RESULTS

### Diagnostic, Demographic, and Epidemiologic Findings

Of the 60 pediatric patients recruited for this study, 35 had confirmed scrub typhus infection. The PCR result was positive in 31 patients, whereas with the IFA, 34 patients had a detectable scrub typhus group IgM titer higher than the specified cutoffs. We found good concordance between the diagnostic assays; 30 (97%) of the 31 PCR-positive patients were found also to be positive for scrub typhus IgM. All 39 controls for whom a sample was available tested negative for scrub typhus (an insufficient amount of blood was collected for 1 control).

Demographic and exposure history data are shown in Supplementary Table 1. The controls were older and more likely to identify as Thai, whereas the patients were more likely to be younger, identify as belonging to a hill tribe, and have had more contact with chickens and hilly areas before admission. Patients who belonged to a hill tribe (31 of 35 [89%]) were mostly Akha (20 of 31 [65%]) or Lahu (7 of 31 [23%]), and the Karen, Hmong, Lisu, and Yao or Mien tribes contributed 1 (3%) of 31 patients each. Hill-tribe distribution was less skewed in the controls (Karen, 9 of 24 [38%]; Akha, 8 of 24 [33%]; Yao or Mien, 4 of 24 [17%]; Lahu, 3 of 24 [13%]; unknown, 1 of 24 [4%]; and both Karen and Akha, 1 of 24 [4%]).

The majority of patients with scrub typhus were from the western, rural and mountainous region of the province, and we found some clustering, suggestive of multiple high-transmission foci. A larger proportion of patients were admitted between June and November than between December and May (33 and 2 patients, respectively) (0.94 [95% CI, 0.87 to 1.02] and 0.06 [95% CI, −0.02 to 0.13], respectively; *P* < .001). This period coincided with the wet and early winter seasons, in northern Thailand.

### Clinical, Laboratory, and Radiologic Features

In Table 1, clinical features of the patients on admission are shown. All the patients had a fever or history of fever before admission. Patients who were admitted directly to the provincial hospital were febrile for a shorter period of time (6 days [IQR, 3–9 days]) than those who were transferred from another hospital (10 days [IQR, 7–11 days]) (*P* = .006). Other common findings included the presence of an eschar (60%), cough (60%), lymphadenopathy (43%), headache (40%), rash (34%), vomiting (31%), and hepatomegaly (31%). Eschars were detected most frequently on the external genitalia (exclusively in boys) and the head and inguinal regions. A temperature of >37.5°C (31 of 35 [89%]), tachypnea (16 of 35 [46%]), and tachycardia (11 of 35 [31%]) were common vital sign features on admission.

**Table 1.** Clinical, Laboratory, and Radiographic Features on Admission of Pediatric Patients With Scrub Typhus and Comparative Laboratory Results for the Healthy Control Group

Clinical Feature	Patients With ST (n = 35)	Healthy ST-Exposed Controls (n = 40)	*P * ^a^
Length of hospital stay (median [IQR]) (days)	5 (4–7)	—	—
Duration of fever before admission (median [IQR]) (days)	7 (5–10)	—	—
Transferred from another hospital (n [%])	14 (40)	—	—
Fever (>37.5°C on or during admission) (n [%])	35 (100)	—	—
Temperature on admission (median [IQR]) (°C)	38.9 (38.0–39.5)	—	—
Rigors (n [%])	5 (14)	—	—
Eschar (n [%])	21 (60)	—	—
Eschar location (n [%])		—	—
External genitalia	9 (43)	—	—
Head	4 (19)	—	—
Inguinal region	3 (14)	—	—
Chest	2 (10)	—	—
Axillary region	1 (5)	—	—
Neck	1 (5)	—	—
Shoulder	1 (5)	—	—
Buttock	1 (5)	—	—
Waistline	1 (5)	—	—
Rash (n [%])	12 (34)	—	—
Rash type (n [%])		—	—
Petechial	5 (42)	—	—
Maculopapular	5 (42)	—	—
Macular	2 (17)	—	—
Cough (n [%])	21 (60)	—	—
Dyspnea (n [%])	6 (17)	—	—
Lung crepitations (n [%])	6 (17)	—	—
Intubated on or within 24 h of admission (n [%])	5 (14)	—	—
Epistaxis (n [%])	1 (3)	—	—
Hemoptysis (n [%])	0 (0)	—	—
Nausea (n [%])	3 (9)	—	—
Vomiting (n [%])	11 (31)	—	—
Diarrhea (n [%])	10 (29)	—	—
Abdominal pain (n [%])	7 (20)	—	—
Jaundice (n [%])	0 (0)	—	—
Hepatomegaly (n [%])	11 (31)	—	—
Splenomegaly (n [%])	3 (9)	—	—
Gum bleeding (n [%])	0 (0)	—	—
Hematemesis (n [%])	0 (0)	—	—
Conjunctivitis (n [%])	8 (23)	—	—
Subconjunctival hemorrhage (n [%])	2 (6)	—	—
Retro-orbital pain (n [%])	1 (3)	—	—
Tinnitus (n [%])	0 (0)	—	—
Deafness (n [%])	0 (0)	—	—
GCS (median [IQR])	15 (15–15)	—	—
Headache (n [%])	14 (40)	—	—
Neck stiffness (n [%])	3 (9)	—	—
Seizures (n [%])	3 (9)	—	—
Confusion (n [%])	3 (9)	—	—
Vertigo (n [%])	1 (3)	—	—
Arthralgia (n [%])	0 (0)	—	—
Myalgia (n [%])	4 (11)	—	—
Lymphadenopathy (n [%])	15 (43)	—	—
Hemoglobin (median [IQR]) (g/dL)	11.3 (10.3–11.9)	13.2 (12.5–14.1)	<.001^b^
Hematocrit (median [IQR]) (%)	34.4 (31.7–36.7)	40.8 (38.8–43.8)	<.001^b^
Platelets (median [IQR]) (×10^3^/mm^3^)	107 (58–178)	290 (256–355)	<.001^b^
White blood cell count (median [IQR]) (×10^3^/mm^3^)	8.1 (5.9–11.5)	8.0 (6.9–9.7)	.733
Neutrophils (median [IQR]) (×10^3^/mm^3^)	4.2 (3.1–7.2)	4.7 (3.2–5.5)	.742
Lymphocytes (median [IQR]) (×10^3^/mm^3^)	1.7 (1.3–3.4)	2.7 (2.1–3.1)	.024^b^
Monocytes (median [IQR]) (×10^3^/mm^3^)	0.5 (0.2–0.7)	0.6 (0.4–0.7)	.357
Eosinophils (median [IQR]) (×10^3^/mm^3^)	0.0 (0.0–0.0)	0.2 (0.1–0.5)	<.001^b^
Blood urinary nitrogen (median [IQR]) (mg/dL)	9.8 (7.1–11.6)	10.0 (9.4–11.8)	.345
Creatinine (median [IQR]) (mg/dL)	0.43 (0.37–0.55)	0.52 (0.42–0.59)	.146
Sodium (median [IQR]) (mmol/L)	131 (129–134)	—	—
Potassium (median [IQR]) (mmol/L)	3.5 (3.3–4.0)	—	—
Chloride (median [IQR]) (mmol/L)	100 (97–105)	—	—
Globulin (median [IQR]) (g/dL)	3.1 (2.6–3.5)	3.2 (3.0–3.6)	.120
Albumin (median [IQR]) (g/dL)	2.9 (2.6–3.3)	4.4 (4.2–4.5)	<.001^b^
Bilirubin direct (median [IQR]) (mg/dL)	0.2 (0.1–0.2)	0.2 (0.1–0.2)	.508
Bilirubin total (median [IQR]) (mg/dL)	0.4 (0.3–0.5)	0.4 (0.3–0.6)	.799
AST (median [IQR]) (IU/L)	140 (78–203)	23 (19–28)	<.001^b^
ALT (median [IQR]) (IU/L)	69 (44–111)	14 (11–18)	<.001^b^
ALP (median [IQR]) (IU/L)	162 (107–224)	229 (159–310)	.032^b^
CRP (median [IQR]) (µg/mL)	44 (22–88)	<5 (<5–<5)	<.001^b^
CXR performed (n [%])	21 (60)	—	—
CXR findings		—	—
Normal (n [%])	12 (57)	—	—
Pulmonary infiltrates (n [%])	7 (33)	—	—
Pulmonary infiltrates and edema (n [%])	2 (10)	—	—

Abbreviations: ALP, alkaline phosphatase; ALT, alanine aminotransferase; AST, aspartate aminotransferase; CRP, C-reactive protein; CXR, chest radiography; GCS, Glasgow Coma Scale; IQR, interquartile range; ST, scrub typhus.

^a^The analysis was performed using the Mann–Whitney U test.

^b^Statistically significant result.

Laboratory and CXR results for patients and available laboratory results for the controls are also listed in Table 1. The patients with scrub typhus had lower platelet, lymphocyte, and eosinophil counts than the controls. Although the patients also had lower hemoglobin and hematocrit levels, these findings might be confounded by the age difference between the groups. Elevated aspartate aminotransferase (AST), alanine aminotransferase, and C-reactive protein levels along with low albumin and alkaline phosphatase levels were the main biochemical findings in the patients. In patients for whom CXR results were available, normal appearances (12 of 21 [57%]) and pulmonary infiltrates (9 of 21 [43%]) were the main findings. Other microbiological tests performed at the discretion of local clinicians (blood, urine, stool, sputum, and cerebrospinal fluid cultures; malaria film; dengue and leptospirosis RDTs) all yielded negative or nonsignificant results.

### Treatments, Complications, and Outcomes

The treatments given are outlined in Supplementary Table 2. Preadmission antibiotics were given to 22 (63%) of the 35 patients, but only 4 (18%) of these 22 patients received appropriate antibiotics for scrub typhus (chloramphenicol, doxycycline, azithromycin, or rifampicin). In contrast, all the patients received antibiotics effective against scrub typhus during their admission, which is a testament to the experience and awareness of local pediatricians at the provincial hospital. Doxycycline was used in 30 (86%) of 35, chloramphenicol in 14 (40%) of 35, azithromycin in 3 (9%) of 35, and rifampicin in 3 (9%) of 35 patients.

A large proportion of the patients (14 of 35 [40%]) developed a complication, as outlined in Table 2; most of these patients (11 of 14 [79%]) had more than 1 systemic complication of scrub typhus with a degree of overlap between the complications observed. For example, 3 patients with pneumonitis developed acute respiratory distress syndrome, and 1 patient had acute myocarditis that progressed to circulatory shock. Despite the severe nature of scrub typhus in this study, the majority of patients recovered; 1 patient with multiorgan failure died.

**Table 2.** Complications Observed in Patients With Scrub Typhus

Complication	Patients With Complication (n [%])
Severe hepatitis^a^	9 (26)
Severe thrombocytopenia^b^	7 (20)
Pneumonitis	5 (14)
Circulatory shock^c^	4 (11)
Acute respiratory distress syndrome^d^	3 (9)
Acute kidney injury^e^	2 (6)
Hematologic abnormalities consistent with DIC^f^	2 (6)
Meningitis^g^	2 (6)
Meningoencephalitis^h^	1 (3)
Myocarditis^i^	1 (3)
Upper gastrointestinal hemorrhage	1 (3)

Abbreviation: DIC, disseminated intravascular coagulation.

^a^Aspartate aminotransferase or alanine aminotransferase level ≥5 times the upper limit of normal.

^b^Platelet count of <50 × 10^3^/mm^3^.

^c^Severe circulatory failure that required inotropic and vasopressive support.

^d^Acute respiratory failure, hypoxia, and bilateral infiltrates seen with chest radiography.

^e^Clinically denoted (reduced urine output) (elevated creatinine level in 1 case).

^f^Hematologic abnormalities consistent with DIC, tentative diagnosis based on thrombocytopenia, prolonged prothrombin time, and prolonged activated partial thromboplastin time.

^g^Clinical features (meningism), cerebrospinal fluid leukocytosis in 1 case.

^h^Clinical features (confusion, disorientation, and visual hallucination), cerebrospinal fluid leukocytosis.

^i^Reduced ejection fraction and biventricular enlargement on transthoracic echocardiogram, elevated troponin I level.

**Figure 1.**
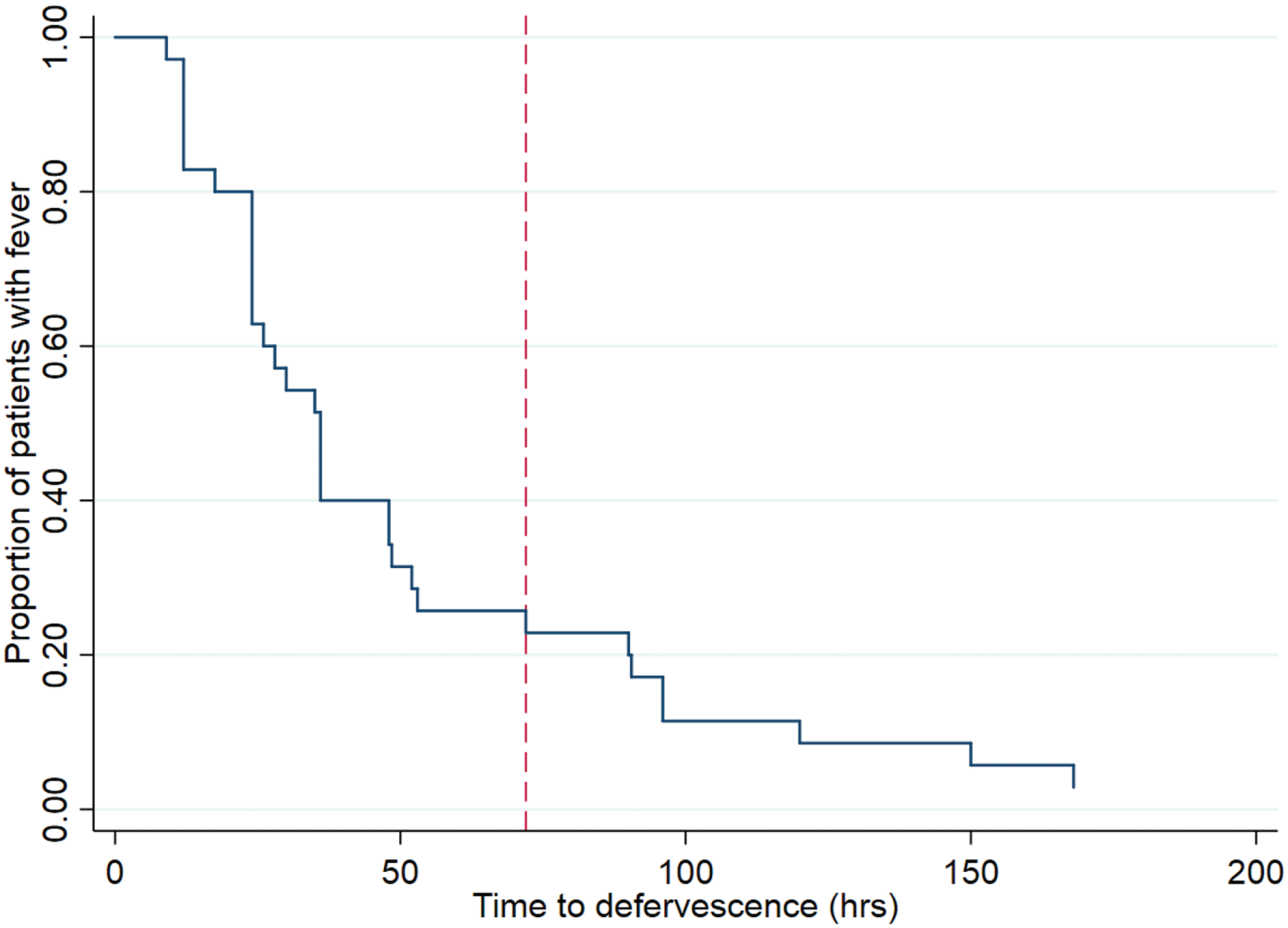
Kaplan–Meier survival curve for fever clearance. The vertical dashed line indicates the 72-hour fever-clearance time treatment-failure cutoff.

The median FCT for this pediatric scrub typhus cohort was 36 hours (IQR, 24–53 hours), and the median headache-clearance time was 36 hours (IQR, 24–48 hours). The proportion of febrile patients over time is shown in Figure 1. Treatment failure, based on prolonged FCT, was observed in 8 (23%) of 35 patients, 7 of whom had unresolved fever and persisting complications and 1 of whom died. The mortality rate was 3% in this study. The majority of patients completed the study (33 of 35 [94%]); 1 patient died and 1 patient missed the 52-week follow-up because of migration. We encountered no clinical or laboratory-confirmed (using PCR and IFA) case of relapse or reinfection during the entire follow-up period.

Univariate logistic regression analysis was performed to determine variables associated with treatment failure (results are listed in Table 3). During the study period, chloramphenicol was exclusively administered intravenously in patients, which might have selected for those with severe disease (ie, those who were intubated and unable to take oral antibiotics), which increased the likelihood of treatment failure. In contrast, we found no evidence of treatment failure associated with doxycycline use during admission (only oral formulations were available); the use of doxycycline was associated with treatment success (odds ratio, 0.1 [95% CI, 0.0–1.0]; *P* = .052). Hepatomegaly, higher total bilirubin level, and severe hepatitis (mainly raised AST level [≥5 times the upper limit of normal]) were also significantly associated with treatment failure. Multivariate logistic regression analysis was performed by using severe hepatitis as the representative hepatic variable (to minimize the risk of multicollinearity) along with transfer from another hospital and chloramphenicol use during admission (Table 3). Severe hepatitis remained significantly predictive of treatment failure in this model.

**Table 3.** Variables Significantly Associated With Treatment Failure in Logistic Regression Analyses^a^

Variable	OR	95% CI	*P*
Univariate logistic regression analysis			
Transferred from another hospital	7.1	1.2–43.1	.033^c^
Hepatomegaly	13.2	2.0–85.8	.007^c^
Severe hepatitis^b^	24	3.2–177.4	.002^c^
Bilirubin total (mg/dL)	106.6	2.2–5191.9	.019^c^
Chloramphenicol use during admission	20	2.1–192.7	.010^c^
Multivariate logistic regression analysisa			
Transferred from another hospital	2.7	0.2–29.1	.420
Severe hepatitis	13.1	1.4–119.9	.023^c^
Chloramphenicol use during admission	7.6	0.6–102.7	.128

Abbreviations: CI, confidence interval; OR, odds ratio.

^a^Variables included in the multivariate logistic regression analysis limited to “transferred from another hospital,” “chloramphenicol use during admission,” and “severe hepatitis” as the representative hepatic variable to minimize the risk of multicollinearity. Odds ratio for the multivariate log regression analysis is adjusted OR.

^b^Defined as an aspartate aminotransferase or alanine aminotransferase level of ≥5 times the upper limits of normal.

^c^Statistically significant result.

## DISCUSSION

In this report, we describe a well-characterized cohort of pediatric patients with scrub typhus, according to robust diagnostic criteria, and a comparatively healthy control group with a prolonged follow-up period of 1 year in northern Thailand. Scrub typhus occurs more often in rural mountainous areas and disproportionately affects the hill-tribe population (particularly the Akha and Lahu). Disease seasonality reflects previous findings in adults from the same province [33]. An eschar was present in 60% of our patients, similar to rates previously reported, which reflects the absent or limited preexisting immunity in children compared to that in adults [10, 13–15]. Cough, tachypnea, and lymphadenopathy in Thai children with scrub typhus have been reported previously, although hepatomegaly was less common in our cohort [14, 15, 17]. Thrombocytopenia, a raised C-reactive protein level, a low albumin level, and elevated hepatic enzyme levels (particularly AST) were common findings; the latter was shown previously to be useful for differentiating scrub typhus from other causes of undifferentiated fever, such as dengue, in adults [33, 34].

The accuracy of the RDT used for screening in this pediatric study was disappointing; many false-positive results occurred despite promising results when it was validated using adult samples from Chiang Rai [25]. The development of an accurate, cost-effective diagnostic test, validated for adults and children throughout the region of endemicity, clearly remains a key diagnostic goal.

Appropriate antibiotics were administered once the patients arrived at the provincial hospital. However, antibiotics used in the community and other healthcare facilities frequently excluded coverage for scrub typhus and other rickettsial diseases, an observation also made in adults [33]. As a consequence, effective treatment was delayed in many patients, which might have contributed to the high complication (40%) and treatment failure (23%) rates in our study. Disease awareness among healthcare workers and the general population throughout the region must improve. Markers of hepatic dysfunction, previously shown to be associated with disease severity, were significantly associated with treatment failure in our study [34]. Specifically, severe hepatitis, as defined by a markedly elevated AST or alanine aminotransferase level (ALT), might be predictive of treatment failure in children.

It is likely that factors beyond delays in recognition and treatment contributed to the high treatment failure rates. Bacterial factors, such as the diversity of virulence of infecting *O. tsutsugamushi* strains, have been described [6, 35]. Tentative findings of antibiotic resistance from northern Thailand have been reported and widely cited but have yet to be verified independently [12]. Indeed, recent in vitro antibiotic-susceptibility testing of allegedly resistant *O. tsutsugamushi* strains failed to provide supportive evidence of resistance [36]. Improving our understanding of the virulence of *O. tsutsugamushi* and its infective life cycle and interstrain variability is crucial for solving the decades-old conundrum of treatment failure in patients with scrub typhus infection. Recent studies have begun to explore these fundamentals [37, 38].

Pharmacologic factors, including the mechanisms of action and pharmacokinetics of the antibiotics used for scrub typhus, also contribute to treatment outcome [39]. Specifically, drug concentrations achieved within cells (such as peripheral blood mononuclear cells) with current antibiotic doses warrant further study [40]. Host immunity has been shown to be protective in humans and nonhuman primates [10, 11]. However, immunity is short-lived; protection from heterologous and homologous strains is limited to a few months and 1 to 2 years, respectively [10, 11]. No vaccine currently provides long-term heterologous protection. Determining the immune correlates of protection will be key to understanding the effect of preexisting immunity on clinical outcome and the development of effective vaccines.

Limitations of this study include the demographic mismatch between the control and patient groups, which occurred despite our efforts to minimize these differences. The patient numbers were relatively small but comparable to those in previous studies on pediatric scrub typhus. Because of limited resources at the time of the study, cultures and susceptibility testing were not performed. In addition, the IFA cutoffs used have not been validated using pediatric samples. However, good concordance between IFA and PCR results in both confirmed and negative cases of scrub typhus suggest that the diagnostic criteria we used were appropriate for this population and region.

## CONCLUSION

Scrub typhus remains a severely neglected infectious disease despite growing evidence of its importance in the rural tropics. Previous studies in children have been limited by absent or short follow-up, lack of a control group, and reliance on unvalidated diagnostic tests. The results of our study show the value of using diagnostic criteria that combine both validated bacterial and antibody-based assays to greatly increase the certainty of diagnosis. Pediatric scrub typhus infection in northern Thailand is often severe and potentially fatal. Additional studies of the bacterial, pharmacologic, and immunologic factors related to treatment outcome, along with measures to improve public awareness of this disease, should be prioritized.

## Supplementary Material

piz014_suppl_Supplementary_Table-S1

piz014_suppl_Supplementary_Table-S2

## Notes

***Acknowledgments.*** We thank the study participants, patients, and staff at Chiang Rai Prachanukroh Hospital. In particular, we thank the deputy director Achara Laongnualpanich for her support. We are grateful to the study nurses, Suthathip Kaewta, Nidanuch Tasak, and Piangnet Jaiboon, for their help with the study and Areerat Thaiprakhong, Nattapon Pinthong, Manutsanun Sumonwiriya, Tippawan Anantatat, and Ampai Tanganuchitcharnchai for laboratory support. We are indebted to the Clinical Trial Support Group staff at Mahidol-Oxford Tropical Medicine Research Unit, including Noppawan Lunjarungsimalert, Prayoon Yuentrakul, Pasathorn Sirithiranont, and Zoe Doran. Last, we thank Mavuto Mukaka for statistical support.

***Financial support.*** This work was supported by the Wellcome Trust as part of the Mahidol-Oxford Tropical Medicine Research Unit Tropical Medicine Research Programme and the Li Ka Shing Foundation Global Health Programme at the University of Oxford (strategic award LG34 to D. H. P.).

***Potential conflicts of interest.*** The RDTs used in this study were provided free of charge by the manufacturer, who did not participate in the design or completion of the study, had no access to the study data, and was not involved in data analysis or the writing of the manuscript before submission for publication. All authors: No reported conflicts of interest. All authors have submitted the ICMJE Form for Disclosure of Potential Conflicts of Interest. Conflicts that the editors consider relevant to the content of the manuscript have been disclosed.
