# Supplementary material for: Clinical Characteristics and Outcome of Children Hospitalized With Scrub Typhus in an Area of Endemicity
Source: J Pediatric Infect Dis Soc. 2019 Mar 13;9(2):202–9. doi: 10.1093/jpids/piz014 (PMC7192406; doi:10.1093/jpids/piz014)
Supplement: piz014_suppl_Supplementary_Table-S1 [file piz014_suppl_supplementary_table-s1.docx]

**Table S1.** Demographic and exposure history among scrub typhus paediatric patients and healthy controls from the same endemic region.

|  | **Patients (STP),**  **n = 35** | **Healthy controls (STE), n = 40** | ***p* value** |
| --- | --- | --- | --- |
| Age (years; median, IQR) | 6 (3-10) | 11.5 (8.5-13.5) | *<0.001* |
| Sex:   - Male, n (%) - Female, n (%) | 24 (69%)  11 (31%) | 21 (53%)  19 (48%) | 0.156 |
| Ethnicity:   - Thai, n (%) - Hill tribe, n (%) - Lao, n (%) | 21 (60%)  31 (89%)  1 (3%) | 38 (95%)  24 (60%)  0 (0%) | *<0.001*  *0.008*  0.467 |
| Student, n (%) | 29 (83%) | 38 (95%) | 0.136 |
| Insect exposure:   - Flea, n (%) - Mite, n (%) - Tick, n (%) - Lice, n (%) | 8 (23%)  3 (9%)  3 (9%)  1 (3%) | 13 (33%)  6 (15%)  10 (25%)  1 (3%) | 0.353  0.489  0.073  1.000 |
| Animal exposure:   - Rat, n (%) - Cat, n (%) - Dog, n (%) - Pig, n (%) - Cow, n (%) - Horse, n (%) - Rabbit, n (%) - Chicken, n (%) - Duck, n (%) - Bird, n (%) - Fish, n (%) - Squirrel, n (%) - Snake, n (%) | 25 (71%)  27 (77%)  25 (71%)  14 (40%)  2 (6%)  1 (3%)  1 (3%)  32 (91%)  2 (6%)  0 (0%)  0 (0%)  0 (0%)  0 (0%) | 21 (53%)  27 (68%)  30 (75%)  14 (35%)  1 (3%)  0 (0%)  1 (3%)  26 (65%)  0 (0%)  4 (10%)  1 (3%)  2 (5%)  1 (3%) | 0.093  0.353  0.727  0.655  0.596  0.467  1.000  *0.011*  0.214  0.118  1.000  0.495  1.000 |
| Visit place:   - Rice field, n (%) - Garden, n (%) - Forest/jungle, n (%) - Hill, n (%) - Valley, n (%) - Corn field, n (%) - Tea plantation, n (%) - Palm plantation, n (%) | 17 (49%)  27 (77%)  18 (51%)  25 (71%)  5 (14%)  2 (6%)  1 (3%)  0 (0%) | 22 (55%)  23 (58%)  17 (43%)  13 (33%)  6 (15%)  0 (0%)  0 (0%)  1 (3%) | 0.578  0.072  0.439  *0.001*  0.930  0.214  0.467  1.000 |
| History of scrub typhus, n (%) | 0 (0%) | 0 (0%) | - |

[STP – Scrub Typhus Patients, STE – Scrub Typhus Exposed healthy controls; exposure in the STP group was in the preceding 2 weeks prior to admission; subjects may have more than 1 ethnicity (e.g. Thai and hill tribe); analysis performed using Pearson’s Chi-squared or Fisher’s exact test as appropriate]
