# Supplementary material for: Clinical Characteristics and Outcome of Children Hospitalized With Scrub Typhus in an Area of Endemicity
Source: J Pediatric Infect Dis Soc. 2019 Mar 13;9(2):202–9. doi: 10.1093/jpids/piz014 (PMC7192406; doi:10.1093/jpids/piz014)
Supplement: piz014_suppl_Supplementary_Table-S2 [file piz014_suppl_supplementary_table-s2.docx]

**Table S2.** Treatment of scrub typhus patients.

| **Treatment** | **Scrub typhus patient group (STP), n = 35** |
| --- | --- |
| Pre-admission antibiotics, n (%) | 22 (63%) |
| Antibiotics (n=22):   - Amoxicillin, n (%) - Ceftriaxone, n (%) - Ceftriaxone with scrub typhus active antibiotics, n (%) - Ceftriaxone with amoxicillin or ampicillin, n (%) - Unknown, n (%) | 8 (36%)  4 (18%)  4 (18%)  2 (9%)  4 (18%) |
| Admission antibiotics, n (%) | 35 (100) |
| Antibiotics (n=35):   - 3^rd^ generation cephalosporin and scrub typhus active antibiotics*, n (%) - Doxycycline alone, n (%) - Doxycycline and chloramphenicol, n (%) - Doxycycline and ampicillin, n (%) - Chloramphenicol and azithromycin, n (%) | 16 (46%)  15 (43%)  2 (6%)  1 (3%)  1 (3%) |
| Steroids, n (%) | 5 (14%) |

* In this group (16 patients), 11 patients received chloramphenicol while 5 did not.

[Scrub typhus active antibiotics include doxycycline, chloramphenicol, azithromycin and rifampicin; 3^rd^ generation cephalosporin mainly represented by ceftriaxone but also include cefotaxime and ceftazidime; steroids include dexamethasone and hydrocortisone]
